# A combinatorial approach implementing new database structures to facilitate practical data curation management of QTL, association, correlation and heritability data on trait variants

**DOI:** 10.1093/database/baad024

**Published:** 2023-04-21

**Authors:** Zhi-Liang Hu, Carissa A Park, James M Reecy

**Affiliations:** Department of Animal Science, Iowa State University, 2255 Kildee Hall, 806 Stange Road, Ames, IA 50011-3150, USA; Department of Animal Science, Iowa State University, 2255 Kildee Hall, 806 Stange Road, Ames, IA 50011-3150, USA; Department of Animal Science, Iowa State University, 2255 Kildee Hall, 806 Stange Road, Ames, IA 50011-3150, USA

## Abstract

A precise description of traits is essential in genetics and genomics studies to facilitate comparative genetics and meta-analyses. It is an ongoing challenge in research and production environments to unambiguously and consistently compare traits of interest from data collected under various conditions. Despite previous efforts to standardize trait nomenclature, it remains a challenge to fully and accurately capture trait nomenclature granularity in a way that ensures long-term data sustainability in terms of the data curation processes, data management logistics and the ability to make meaningful comparisons across studies. In the Animal Quantitative Trait Loci Database and the Animal Trait Correlation Database, we have recently introduced a new method to extend livestock trait ontologies by using trait modifiers and qualifiers to define traits that differ slightly in how they are measured, examined or combined with other traits or factors. Here, we describe the implementation of a system in which the extended trait data, with modifiers, are managed at the experiment level as ‘trait variants’. This has helped us to streamline the management and curation of such trait information in our database environment.

**Database URL** https://www.animalgenome.org/PGNET/

## Introduction

**Figure 1.**
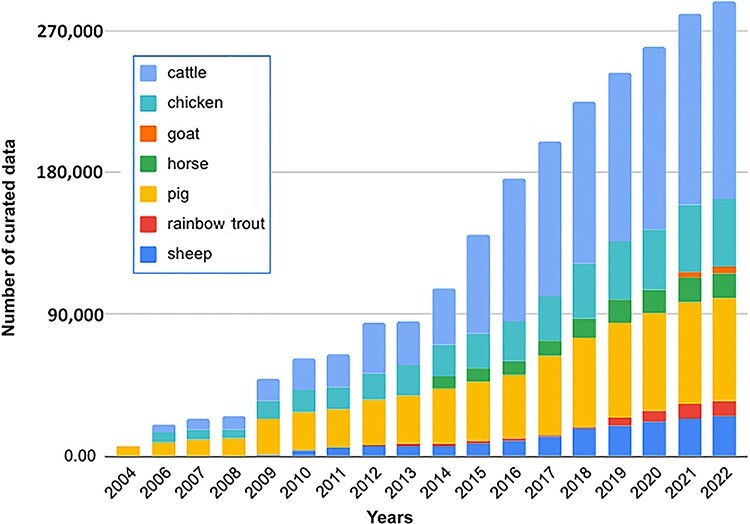
An increase in curated QTL/association/correlation/heritability data in the Animal QTLdb and CorrDB over the past 18 years. The data are plotted using log-transformed values for better visualization.

The Animal Quantitative Trait Loci Database (QTLdb; https://www.animalgenome.org/QTLdb) and Animal Trait Correlation Database (CorrDB; https://www.animalgenome.org/CorrDB) are actively curated repositories for the collection of published livestock genetic data in electronic form for easy search and comparison across studies. The two databases were originally developed separately, and joint development efforts were undertaken for parts they share in common, such as livestock trait management. The QTLdb houses QTL, single nucleotide polymorphism and phenotype association data, and the CorrDB houses trait correlation and heritability data, published in the past 20+ years for multiple livestock animal species (1). The data volume increase in these databases has been phenomenal. For example, the amount of cattle QTL and the association data curated into the Animal QTLdb has undergone a 359-fold increase over the past 18 years (Figure 1), owing to explosive growth in the published data brought on by continued progress in sequencing and genotyping technologies. Both databases have been widely used by people from >40 countries. For example, in 2022, the databases received over 5 million web visits by 51 000 unique users, which generated >90-GB data downloads. Since the data curated into the database are from thousands of scientific papers published in >200 journals, it has been a continual challenge to develop, improve and maintain a sustainable database structure, and the curation tools required not only to ensure data integrity and consistency but also to allow data reported in different formats and levels of granularity to be translated into a common form for across-the-board comparisons. One such challenging area is trait information curation and management.

To meet these challenges, we first introduced the use of controlled vocabularies into the QTLdb to manage trait description variations using trait categories and trait types (2). Our efforts were further extended by introducing the use of ontologies, which resolve concept meanings and relationships between objects using controlled vocabularies. Currently, we employ the Clinical Measurement Ontology (3), Vertebrate Trait Ontology (4) and Livestock Product Trait Ontology (https://www.animalgenome.org/bioinfo/projects/lpt/). The development and expansion of these ontologies have been undertaken in collaboration with the Medical College of Wisconsin and Mouse Genome Informatics (5, 6) in order to support our daily curation of diverse types of trait information into the database. While these efforts laid the foundation for trait curation and management, capturing variation in trait details still remained a challenge. For example, for the most common trait, ‘Average Daily Gain’ (ADG), there are many methods of recording trait data in different species, including by production stage (e.g. pre-weaning and post-weaning), by population (e.g. cows and heifers), by body weight (e.g. 30–100 kg) or by age/time (e.g. 21–46 days and 6–9 months). The combined use of these factors can dramatically increase the number of required ADG terms in the database, making it difficult to manage them by extending the trait ontology tree structure.

**Figure 2.**
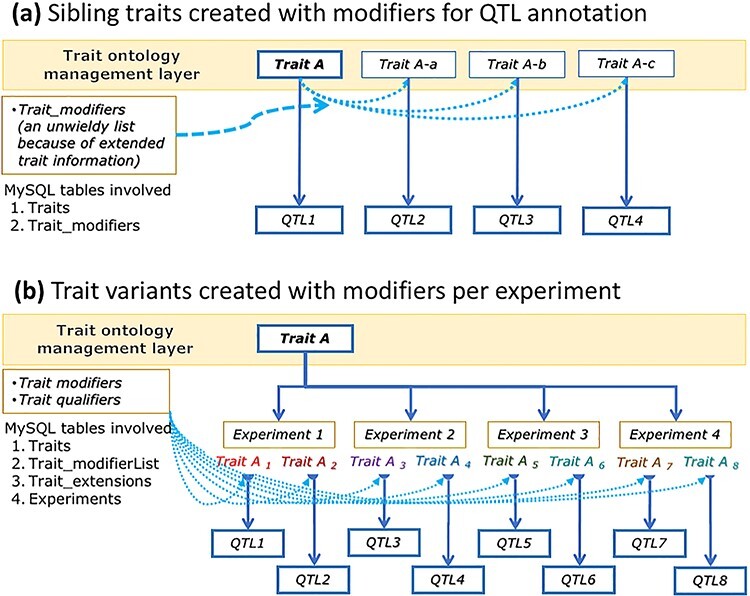
A conceptual graph showing data structure differences between ‘sibling traits’ (modified as part of ontology extensions) (a) and ‘trait variants’ (modified with extended info) created at the experiment level (b), their use in QTL data annotations and their effects on ontology trait data management.

Our previous method for handling such variation was to create ‘sibling traits’ (7), in which the base trait (e.g. ADG) was appended with one or more ‘modifiers’, and the resulting term was added to the trait ontology hierarchy within the database (8) (Figure 2a). When this design was implemented in the QTLdb curator environment, the number of sibling traits quickly became difficult to manage and reuse. Within 2 years, the databases had accumulated as many as 69 sibling traits to ADG, 389 sibling traits to body weight and so on (unpublished data). In addition, we needed to separately maintain a long list of modifiers. These long lists of sibling traits and modifiers were cumbersome to deal with during the curation process, and it was clear that they could soon balloon to unmanageable levels. To address these issues, we took a ‘combinatorial’ approach—partitioning the concepts of traits, where applicable, to identify common characteristics for compartmentalized data management. We have developed a ‘trait variant’ structure for the practical management of trait data in the Animal QTLdb and CorrDB curator tools environments. Here, we report our initial success with this effort.

## Results

This work involves (i) the development of a curation route and database structure for the new ‘trait variant’ management and (ii) the migration of existing data from the previous ‘sibling traits’ to the new ‘trait variant’ data curation and management structure.

### Concepts

A trait term may be ‘modified’ by a property, or ‘descriptor’, such as an experimental or environmental factor, to produce an extended version of the trait term as a new term. Examples of such extensions are intramuscular fat content (IMF) measured in different muscles (e.g. gluteus medius and longissimus) or using different methods (e.g. ultrasound and petroleum ether extraction); or milk yield (MY) measured daily or over the entire lactation period or compared between the first and second lactations. For these examples, IMF and MY are the base traits, the additional properties are modifiers and the extended names of the base traits plus modifiers are trait variants. In order to compartmentalize the modifier terms for a controlled list, we introduced qualifiers to further define the use of a modifier. This effectively partitioned the ‘modifiers’ used previously in ‘sibling traits’ into two parts: modifiers and qualifiers.

### Implementation

**Figure 3.**
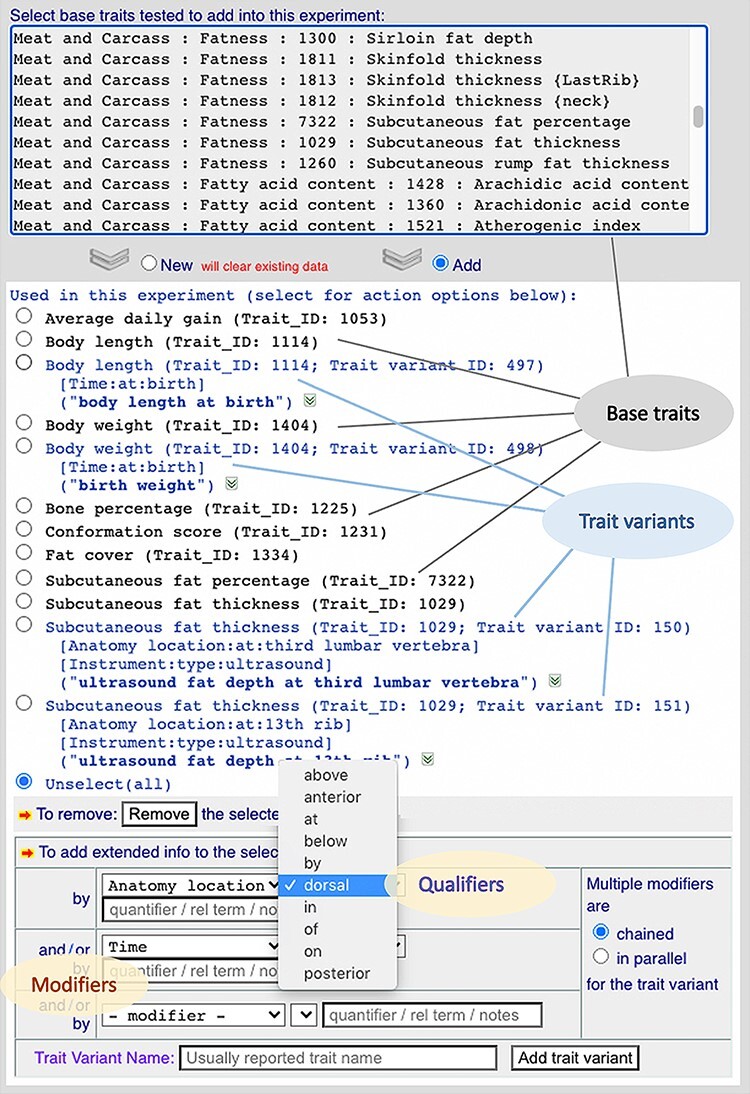
A screenshot of a curation web form showing part of the experiment curation environment. It shows how this implementation allows trait variants to be created from their base traits using controlled vocabulary lists to define modifiers/qualifiers.

By partitioning modifiers from qualifiers, we were able to create a modifier list with a smaller number of categories (analysis, anatomy location, environment, population, instrument, measurement, parity, kinship, stage, time and treatment) for a drop-down menu in the curator panel (Figure 3). Each of the modifiers has an accompanying list of qualifiers (Table 1). These modifiers and qualifiers are used as controlled vocabularies in the curation environment. Additional free-text terms can be added to further define a modifier (e.g. ‘6 months’ for time/age). Our implementation of the trait annotation using modifiers/qualifiers at the experiment level has helped us speed up the curation process by structuring the data complexities into the database and employing programs to semi-automate the processes of data alignment. A real-time summary of curated trait variants (https://www.animalgenome.org/QTLdb/doc/meta/tvarinfo) provides a reference for curators as well as a review of curated data by (base) trait.

**Table 1.** ‘Modifiers’ and ‘qualifiers’ used in the implementation of a new trait variant management system, where trait variants are curated at the experiment level

	Modifiers	Qualifiers
1	Analysis	Adjusted, calculated and estimated
2	Anatomy location	Above, anterior, at, below, by, dorsal, in, of, on and posterior
3	Environment	Challenge, confinement and stress
4	Population	Calves, cows, ewes, heifers, layers and sows
5	Instrument	Manufacturer, name and type
6	Measurement	Amount, area, character, color, composition, count, length, maximum, response, speed and weight
7	Parity	Count
8	Kinship	Dam, daughter, maternal, paternal and sire
9	Stage	Adult, end, feeder, finisher, gestation, lactation, nursing, parturition, start, weaning and yearling
10	Time	After, age, at, basis, before, by, duration and weight
11	Treatment	Challenge, drug, fast, feed, freeze, thaw and trim

This scheme helped relieve curation and data management burdens caused by long and unwieldy lists of ‘sibling traits’. In addition to these modifiers and qualifiers as controlled vocabularies, we also have a free-text field to allow additional descriptions when the modifier/qualifier does not precisely cover the scenario. The data collected with this free-text field will be used to improve the controlled list of modifiers/qualifiers.

### Multiple modifiers

With this new management scheme at the experiment level, we are able to maintain minimum numbers of controlled vocabulary terms for modifiers and qualifiers in order to facilitate the consistent use of terms over time. Currently, the system can accommodate up to three (3) modifiers attached to a base trait to cover most, if not all, trait variants we encounter. An example of a trait variant with multiple modifiers is ‘drip loss in pectoralis muscle at 24-hr post-mortem’. If more than one modifier is required to define a trait variant, the new curator tool has a mechanism to denote the relationships between modifiers. For instance, in the example mentioned earlier, the anatomy location (‘in pectoralis muscle’) and time (‘at 24-hr post-mortem’) modifiers are dependent on each other to fully describe the trait; we consider these modifiers linked, or ‘chained’. On the other hand, body weight at weaning could be described either by the stage (weaning) or the age at which it occurs (e.g. 21 days). In this case, ‘at 21 days’ and ‘at weaning’ are independent modifiers (alternatives or ‘in parallel’).

### Improvements

As part of the database transition to using the new curation scheme described earlier, we have begun a migration of all ‘sibling trait’ data curated in previous years to the ‘trait variant’ scheme under the new structure in both the QTLdb and CorrDB. Throughout this transition, a total of 1256 new trait variants have been created for 278 base traits. The new trait variants include 418 for QTL/associations, 425 for correlations and 413 for heritability. This process has affected 22 205 curated data, including 16 227 QTL/associations, 5573 correlations and 415 heritability data (Table 2). As a result of these changes, we have effectively reduced the number of extended trait data managed within the database trait ontology structure by an average of 71.5% for QTL/association/correlation/heritability data in both the QTLdb and CorrDB (Table 3). These results reflect a significant positive impact on the QTLdb and CorrDB, in terms of not only providing a simpler structure for trait concepts but also helping to standardize the curation protocols and setting a sustainable stage for future database developments.

**Table 2.** The number of experiments and annotated data affected in the QTLdb and CorrDB due to trait management changes from ‘sibling traits’ to ‘trait variants’ in 2022

Data	Affected data types	Cattle	Chicken	Goat	Horse	Pig	Rainbow trout	Sheep	Total
QTL/association	Total base traits (BT)	678	370	25	65	692	28	265	**2123**
	BT with variants	28	10	2	1	33	6	13	**93**
	New trait variants	112	114	4	1	110	6	71	**418**
	Experiments affected	123	342	2	1	93	1	39	**625**
	Annotated data affected	10 010	648	10	16	4906	174	463	**16 227**
Correlation	Total BTs	373	106	33	36	252		76	**876**
	BT with variants	42	13		1	17		18	**91**
	New trait variants	181	52		1	101		90	**425**
	Experiments affected	40	21		1	18		22	**102**
	Annotated data affected	1392	135		10	3143		893	**5573**
Heritability	Total BTs	395	112	2	53	285		96	**943**
	BT with variants	43	13	1	1	18		18	**94**
	New trait variants	170	52	3	1	97		90	**413**
	Experiments affected	45	19	1	1	19		22	**107**
	Annotated data affected	163	9	3	1	203		36	**415**

**Table 3.** Total number of trait changes due to the database transition from using ‘sibling traits’ to ‘trait variants’ in 2022

	Sibling traits	Trait variants	Change (%)
QTL/association	2272	418	−81.6
Correlation	902	425	−52.9
Heritability	1061	413	−79.9
Average			−71.5

The successful migration of ‘sibling traits’ to ‘trait variants’ in a relatively short period of time demonstrates that the new data management implementation works well as designed. Furthermore, this implementation has also significantly reduced many of the frustrations of our data curators, as well as database maintainers, regarding the day-to-day work dealing with emerging cases when curating ‘sibling traits’. Allowing trait variants to be curated at the experiment level gives curators the flexibility to address them on a case-by-case basis and helps reduce clutter in the database trait hierarchy while maintaining data stringency at the database level.

From a database management perspective, this work added ‘trait variants’ as an extension to trait ontology terms (‘base traits’), which separates the management of trait variants from the handling of the trait ontology hierarchy (Figure 2b). The addition of MySQL tables in the current implementation (Figure 2a versus Figure 2b) facilitated trait data partitioning, compartmentalization, relationship building and other logistics. To accommodate the data structural changes, web interface tools have been created or updated to facilitate the trait variant curation, integrity checking and data display/download. Overall, these database changes have helped simplify the manual curation of trait nomenclature information, while simultaneously capturing the complexity of published traits.

Appended to base traits, trait variant information is valuable to facilitate data comparisons for end users evaluating data across time and experiments. At the time of this report, we are in the process of making the newly produced trait variant information available in data downloads and web tools. These data will be visible to the public by the April 2023 database release.

## Discussion

Not only does the sheer volume of newly published data create challenges for Animal QTLdb and CorrDB curation, but also curation/database processes must be adapted to accommodate different data formats, new analysis methods and varying levels of trait data granularity. In contrast to our earlier ‘sibling traits’ system, which attempted to add trait variations into a trait ontology and presented extra challenges for ontology development, our method of developing ‘trait variants’ as extensions of ontology terms (‘base traits’) helps isolate complex trait handling outside of trait ontology development. While the concept partitioning method is effective in simplifying the management of complex trait information, we wish to point out that the level of granularity captured needs careful consideration in order to maximize the overall benefits. For example, the need to consider how traits are defined in multiple animal species further increases the level of complexity.

Gkoutos *et al.* (9) demonstrated the use of a decomposition strategy to dissect the terms in the Human Phenotype Ontology into their entity/quality properties using the Phenotype and Trait Ontology. While this was effective in their work using human medical data, it is obvious that more factors are needed for the accurate dissemination of trait information in livestock animals. Our approach using modifiers/qualifiers demonstrates the possibility of partitioning complex traits using additional trait descriptor information and provides a better structure for the curation management of trait details.

Our approach has effectively helped reduce the lengthy list of ‘compound modifiers’, which were impractical to use. (In our previous ‘sibling trait’ management system, trait modifiers were almost developed into a separate ‘ontology’ structure.) While the modifier factor partitioning approach provides possibilities for a more scalable system, it also opens additional opportunities for complex trait curation and management. For example, while we have implemented mechanisms to handle ‘chained’ or ‘parallel’ modifiers, more complex modifier relationships (such as mixed ‘chained’ and ‘parallel’ modifiers) may exist which require solutions in the near future. This is one area in which the current system is still subject to further development to refine the details.

Note that on the trait variant curation form, there is a free-text field (Figure 3) to collect the trait name reported in a publication. This serves to link real-world trait terms used by researchers and/or producers to ontology terms via the trait variant structure and is useful from a data comparison perspective.

Trait ontology development is an ongoing process, and it is expected that the trait variant system will also need to be expanded or updated in the future. It is important to carefully consider the details regarding the implementation of the trait variant system to ensure its ongoing stability and viability. For instance, it is necessary to appropriately distinguish ‘base traits’ and ‘trait variants’. As an example, since 305-day MY is such a widely used measurement standard for bovine dairy production, people may consider it to be synonymous with MY, but there are several other potential modifiers that may apply to the base ‘MY’ trait. In cases like these, there are multiple factors to consider before determining the most appropriate base trait.

Since trait variants are now created and managed at the experiment level, each trait variant must be re-created for every experiment in which it is used. This will be simplified once the patterns of common complex traits partitions/compositions are established. However, it requires curators to be familiar with the commonly reused complex traits or to refer to the established trait variant list for references (https://www.animalgenome.org/QTLdb/doc/meta/tvarinfo). It could be a steep learning curve for new curators, however, necessitating further improvements to the trait curation environment. One possibility is the implementation of an artificial intelligence helper to suggest trait variants and make them easier to introduce. Overall, these changes have not only provided a workable solution for curating complex traits but also given opportunities for further improvements with better-structured data that are more accessible using programs.

## Data Availability

The database contents and tools are all freely available online. QTLdb: https://www.animalgenome.org/QTLdb/; CorrDB: https://www.animalgenome.org/CorrDB/. In addition, the data is also available upon release at several data alliance partner websites, including NCBI: http://www.ncbi.nlm.nih.gov/gene; Ensembl: http://www.ensembl.org/; UCSC: https://genome.ucsc.edu/cgi-bin/hgGateway; Reuters Data Citation Index: http://wokinfo.com/products_tools/multidisciplinary/dci/.
